# Association of Carotenoids intake With Cognitive Impairment : A Cohort Study of Middle‐aged and Elderly People Adults

**DOI:** 10.1002/alz70860_102742

**Published:** 2025-12-23

**Authors:** Gulisiya Hailili, Liyan Huang, Shuang Rong, Changzheng Yuan

**Affiliations:** ^1^ School of Medicine, hangzhou, Zhejiang province, China; ^2^ School of Medicine, Hangzhou, Zhejiang, China; ^3^ The First Affiliated Hospital, Hefei, 230000, China; ^4^ Harvard T. H. Chan School of Public Health, Boston, MA, USA

## Abstract

**Background:**

Previous studies have related associations of carotenoids with cognitive function and Alzheimer's disease. However, evidence regarding the associations of carotenoids with cognitive impairment (CI) is scarce. This study aimed to investigate the relation of dietary intakes of total carotenoids, provitamin A and non‐provitamin A carotenoids to CI among middle‐aged and elderly people.

**Method:**

We conducted a prospective study among US adults aged 50 years and older based on the Health and Retirement Study from 2014 to 2020. Average daily intakes of dietary total carotenoids, provitamin A carotenoids (α‐carotene, β‐carotene and β‐cryptoxanthin) and non‐provitamin A carotenoids (lutein‐zeaxanthin and lycopene) were assessed by a 163‐item semi‐quantitative food frequency questionnaire (FFQ). CI was evaluated using the Langa‐Weir classification. Cox proportional hazards model examined the association between energy‐adjusted carotenoids intake and CI.

**Result:**

Among 5259 participants (mean age of 67.3±9.6 years), the median daily intake of total carotenoids, provitamin A carotenoids and non‐prvitamin A carotenoids were 9.6 mg/d (IQR, 6.8‐13.9), 3.2 mg/d (IQR, 2.0‐5.2) and 6.0 mg/d (IQR, 4.3‐8.6), respectively. After adjustment of sociodemographic characteristics, lifestyle variables, and other dietary factors, the consumption of total carotenoids was found to significantly reduce the risk of CI, with a hazard ratio of 0.81 (95% CI 0.67‐0.97). Furthermore, both provitamin A carotenoids and non‐prvitamin A carotenoids were independently linked to a 19% and 23% lower the risk of incident CI, respectively.

**Conclusion:**

Our findings support a beneficial role of carotenoid consumption on CI in middle‐aged and elderly people, and guiding further investigations into the mechanisms and further validating the cognitive health benefits of a carotenoid‐rich diet.

